# Barriers and facilitators to antidepressant deprescribing – A qualitative interview study with general practitioners in Germany

**DOI:** 10.1080/13814788.2025.2531879

**Published:** 2025-07-24

**Authors:** Jochen Vukas, Vita Brisnik, Linda Sanftenberg, Peter Henningsen, Jochen Gensichen, Tobias Dreischulte

**Affiliations:** ^a^ Institute of General Practice and Family Medicine, LMU University Hospital, LMU Munich, Munich, Germany; ^b^ Graduate Program ‘POKAL ‑ Predictors and Outcomes in Primary Care Depression Care’, Munich, Germany; ^c^ Department of Psychosomatic Medicine and Psychotherapy, University Hospital, Technical University of Munich, Munich, Germany

**Keywords:** Deprescribing, discontinuation, antidepressant, primary care, general practice, qualitative interview study

## Abstract

**Background:**

Long-term use of antidepressants frequently extends beyond clinical guidelines, with limited structured support for deprescribing in primary care. Little is known about the factors that influence general practitioners (GPs) in Germany regarding deprescribing of antidepressants.

**Objectives:**

To identify barriers and facilitators that influence GPs in Germany regarding antidepressant deprescribing. To provide points of departure for developing a targeted intervention to address these challenges.

**Methods:**

We conducted semi-structured interviews with 20 GPs in Bavaria and purposively sampled for diversity in gender and professional experience. The interview topic guide was informed by the Capability-Opportunity-Motivation-Behaviour (COM-B) model and the Theoretical Domains Framework (TDF). Interviews were transcribed verbatim. Thematic analysis was conducted using a structured coding approach.

**Results:**

Key barriers to deprescribing included time constraints, limited practical tools, and inadequate collaboration with specialists, as well as uncertainty about when to deprescribe. Social and psychological factors, such as patient fears, were also significant. Facilitators included strong GP-patient communication, the use of digital tools, pharmacist support, and positive attitudes towards deprescribing.

**Conclusion:**

Antidepressant deprescribing in German primary care is shaped by systemic, social, and behavioural factors. Addressing time constraints, enhancing interdisciplinary collaboration, and integrating decision-support tools into clinical practice could facilitate deprescribing. These insights inform targeted interventions to promote safe and evidence-based antidepressant use. Further research is recommended to develop an intervention suitable for real-world usage.

## KEY MESSAGES

Main barriers to deprescribing antidepressants were: time constraints, lack of digital and practical decision-support tools, limited collaboration with other physicians/disciplines

GPs are motivated and willing to deprescribe antidepressants but require greater technical and social support

Interventions should explore ways to implement appropriate technical (digital) support and increase collaboration with other physicians/disciplines

## Introduction

Although antidepressants play a critical role in managing severe depression and other mental health disorders, their increasing use over recent decades has raised concerns about their application in cases where the risks may outweigh the benefits.

While clinical practice guidelines (CPGs) typically emphasise the effective initiation and intensification of antidepressant treatment, and address some risks of adverse drug reactions (ADRs), they often lack detailed guidance on when and how to attempt deprescribing. This absence of comprehensive recommendations may contribute to the inconsistent implementation of deprescribing practices, resulting in long-term use without regular review [1–4].

In Germany, deprescribing is gaining importance, especially in the context of polypharmacy. While no national policy mandates it, frameworks or CPGs like the NVL Depression recommend regular risk-benefit reviews of antidepressant use. Tools such as the PRISCUS and FORTA lists help identify inappropriate medications in older adults. Initiatives like ‘Choosing Wisely’ and DEGAM guidelines on multimorbidity support medication reviews and discontinuation of non-indicated drugs. The newly established German Deprescribing Network (GerDeN) promotes interdisciplinary collaboration. Together, these efforts form a growing infrastructure to support GPs in making evidence-based deprescribing decisions. In Germany and globally, the majority of patients with common mental health disorders, such as depression and anxiety, are managed in primary care settings [5]. Thus, to support antidepressant deprescribing, it is crucial to understand how general practitioners (GPs) navigate uncertainties around when and how to discontinue these medications, as well as the barriers and facilitators that influence this process.

A recent systematic review from 2024 by van Leeuwen et al. identified 13 studies on healthcare professionals’ perspectives on antidepressant deprescribing; however, none included data from German primary care [6]. This qualitative study aimed to fill this gap as part of a larger project aimed at improving the care of patients with depression in primary care [7].

## Methods

### Study design

We conducted semi-structured interviews with GPs who were purposively sampled for heterogeneity in terms of age and gender. The interview topic guides were informed by the Capability-Opportunity-Motivation-Behaviour model (COM-B) and the linked Theoretical Domains Framework (TDF). The interview data were transcribed verbatim and subjected to qualitative data analysis proposed by Kuckartz und Rädiker [8]. Ethical approval was obtained.

### Sampling and recruitment of general practitioners

We included GPs practising in Bavaria and affiliated with the statutory health insurance system (GKV), because they make up the vast majority of general practices in Germany, in order to ensure structural comparability across care settings and for logistical feasibility. Practices serving exclusively privately insured patients were excluded due to potential differences in treatment patterns and in attitudes towards CPGs, evidence-based medicine, and antidepressant prescribing. GPs currently involved in research on medication safety or geriatric medicine were excluded. Participants were recruited through a network of GP practices affiliated with the Institute of General Practice and Family Medicine at the University Hospital, LMU Munich, Germany.

### Interview topic guide development

We employed two theoretical frameworks to guide our study and to prepare for the use of the BCW (Behaviour Change Wheel model) in a future intervention. The COM-B model helps identify key factors influencing antidepressant deprescribing, categorising them into Capability (psychological and physical ability), Opportunity (external facilitators or barriers), and Motivation (cognitive drivers of behaviour) [9]. The Theoretical Domains Framework (TDF) complements this by providing detailed behavioural determinants, including knowledge, skills, social influences, and environmental context [10,11]. The development of topics and questions was informed by a thorough literature review and collaborative discussions among the study team.

### Interview procedure

All 20 one-to-one interviews were conducted in participants’ offices, typically during lunch breaks or after clinical hours, providing a familiar and comfortable environment conducive to open discussion between the participant and the interviewer. Each session commenced with an overview of the study’s objectives and data protection protocols, followed by obtaining written consent prior to recording. A trained interviewer led all interviews using a flexible, conversational format to encourage spontaneous insights.

### Data collection and transcription

Interviews were audio-recorded and transcribed verbatim by an external transcription service to ensure accuracy. The service adhered to stringent security protocols, including two-factor authentication, to safeguard participant data. Transcriptions followed established scientific conventions, with minor adjustments for fluency, such as smoothing out repetitions and stutters. Data were anonymised during transcription and subsequently uploaded into MAXQDA Analytics Pro (version 24.2.0) for data analysis.

### Data analysis

The data analysis followed a combined inductive-deductive thematic coding approach as outlined by Kuckartz and Rädiker [8], with the hierarchy as follows from lowest to highest level: code, subtheme, theme, category. The process began with familiarisation and code development, where the interviewer reviewed the first three transcripts, created an initial coding tree based on the interview guide, and recorded preliminary impressions through memos. A deductive coding approach was then applied, organising major themes such as barriers and facilitators within the COM-B framework and aligning them with relevant TDF domains. This provided a structured and systematic exploration of the data. Finally, the coded statements were synthesised to identify the most significant barriers and facilitators, with key themes prioritised based on their relevance and frequency. This process ensured a thorough and nuanced understanding of the interview data.

## Results

### Characteristics of participants

Table 1 summarises the study’s participants, comprising 20 general practitioners (11 female, 9 male). They were recruited from Munich (*n* = 8) and the surrounding southwest Bavarian regions, including towns (*n* = 5), small towns (*n* = 4), and villages (*n* = 3). Participants ranged in age from 33 to 64 years, with professional experience between 8 and 37 years. Twelve participants worked in group practices with multiple GPs, and eight (4 female, 4 male) had prior experience of participating in research. One participant withdrew due to personal reasons and was replaced through additional recruitment.

**Table 1.** Characteristics of participants.

No.	Participant ID	Gender	Age	Years of Professional Experience	Region^1^	Single Practice	Study Experience	Interview Duration in Minutes
1	HI.01.01	Male	57	27	City	No	No	39
2	HI.01.02	Female	56	29	City	Yes	Yes	43
3	HI.01.03	Male	51	25	Small town	No	Yes	47
4	HI.01.04	Male	33	8	City	No	No	52
5	HI.01.06	Female	59	29	City	No	Yes	37
6	HI.01.07	Male	46	17	City	Yes	No	39
7	HI.01.08	Female	59	32	City	No	No	40
8	HI.01.09	Female	62	34	City	Yes	No	28
9	HI.01.10	Female	58	29	City	No	No	28
10	HI.02.01	Male	37	10	Small town	No	Yes	54
11	HI.02.02	Male	36	8	Town	No	No	39
12	HI.03.01	Female	55	30	Town	Yes	Yes	26
13	HI.04.01	Female	46	20	Town	Yes	No	42
14	HI.04.02	Female	48	18	Village	Yes	No	25
15	HI.05.01	Male	64	37	Small town	No	Yes	27
16	HI.05.02	Female	46	18	Town	No	No	26
17	HI.06.01	Female	54	27	Town	Yes	No	63
18	ps_01	Male	58	30	Small town	No	Yes	22
19	ps_02	Female	56	30	Village	No	Yes	23
20	ps_03	Male	38	9	Village	Yes	No	16

^1^Differentation by populational size. City: >100,000. Town: ≤100,000. Small town: ≤10,000. Village: ≤1,000. ID: Identification.

### Barriers to antidepressant deprescribing

Illustrative quotes highlighting barriers to antidepressant deprescribing among GPs are presented in Table 2, organised according to the COM-B model and linked to the TDF. The following section provides a detailed description with references to quotes from Table 2.

**Table 2.** Barriers to antidepressant deprescribing reported by general practitioners participating in semi-structured interviews.

TDF domain	Theme	Codes	Sample quotes	No. of GPs reporting codes
COM-B domain: Psychological Capability				**(*n* = 20)**
Knowledge	*Guidance and decision*	Lack of knowledge to CPGs	#1: I: ‘What do you know about guidelines for antidepressants?’ P: ‘Not much, to be honest.’ (HI.04.02)	**9**
Memory, attention and decision processes	*Guidance and decision*	Lack of established routine	#2: ‘In my practice, it is not systematic. Usually, it happens lets say, by chance or when the patient comes in with a specific concern.’ (ps_03)	**13**
			#3 ′Of course, as long-established GPs, we do a lot based on gut feeling – like, how much does someone still need, or what not? We also try, again and again of course, to reduce certain medications, but not that much in everyday practice. That’s why things like techniques, templates or possible applications might be really interesting.’ (HI.01.06)	
COM-B domain: Reflective Motivation				
Beliefs about capabilities	*Guidance and decision*	Uncertainties regarding the decision to deprescribe	#4 ‘It is actually a daily issue, and I would appreciate having more confidence in this area. Perhaps a greater sense of security as well, that, often, we rely on our intuition to determine the dosage, yes? It’s also, finding the right dosage, to be honest, involves a lot of experience, but it is also often a gut feeling about what to prescribe and how much. Sometimes, I wish I had a bit more certainty.’ (HI.01.08)	**9**
			#5 ′But the complete stopping, in quotation marks, is difficult. Because there’s a huge amount of fear, of course also on the patients’ side, about actually leaving it out. And also that all the symptoms might come back. So yes, that’s really difficult, especially with long-term patients. Where I keep asking: ‘Okay, why are you taking this?’ – ‘Well, there was something ten years ago.’ Then I say: ‘Ah yes. And shall we maybe…?’ – ‘No, they don’t know, they’re afraid, they’ve already tried it themselves and the attempt failed.’ And then to build up motivation again, to try it once more – that’s really hard.’ (ps_02)	
	*Interdisciplinarity* *Relationship*	Uncertainties if originally prescribed by general practitioners from other disciplines	#6 ‘I would definitely say so. So, if I had prescribed it myself, lets say, I would feel significantly more confident and independent in deciding to discontinue it.’ (HI.01.07)	**9**
COM-B domain: Automatic Motivation				
Negative reinforcement	*Guidance and decision*	Guidelines, tools, and aids are too complex, time-consuming, not read, and not necessary	#7 ‘I admit, I do not refer to them often because they are too lengthy and not very user-friendly.’ (HI.01.07)	**9**
			#8 ‘I have time to read summaries, short ones. I’ll read long guidelines now and then, if I have a specific question. But really going through them properly – I don’t have the time, and they’re too detailed and too far from everyday practice.’ (HI.01.08)	
			#9 ‘Because I have my own guidelines up here, so I don’t really follow the, well, official guidelines.’ (HI.03.01)	
COM-B domain: Physical Opportunity				
Environmental context and resources	*Time*	Lack of time	#10 ‘Medication plans often include up to 15 different medications. It is nearly impossible for me to review these comprehensively with the patient within the constraints of a typical consultation.’ (HI.01.04)	**17**
	*Guidance and decision*	Lack of practical tools	#11 ‘Not really. We review it periodically and discuss it during team meetings. However, I do not work with the list [PRISCUS] directly on an ad hoc basis because it is simply too cumbersome.’ (HI.05.01)	**12**
			#12 ‘You lay out this and that many tools, and then you end up putting them in a drawer somewhere. The problem is, in general practice you feel like you have to have 100 tools ready at all times, and the ones you actually use tend to be the ones that are already in the system – unfortunately, one has to say.’ (HI.01.03)	
			#13 ‘We usually have it in our heads, but that doesn’t help entirely, because some medications they simply need. And PRISCUS assumes relatively high doses – for example, when mirtazapine or amitriptyline are prescribed. And with low doses, we’ve at least asked the developers, it supposedly no longer applies.’ (ps_01)	
		Lack or inadequacy of guidelines	#14 ‘The main issue is how to prescribe the medications. There is no mention of how to reduce or discontinue them. Additionally, a major weakness of guidelines is that they are often designed for monocausal diseases.’ (HI.01.02)	**10**
	*Reward and acknowledgement*	Financial and economic deficiencies/ disadvantages	#15 ‘And the other component is that you usually do not get compensated if you thoroughly review the medication plan.’ (HI.02.01)	**9**
COM-B domain: Social Opportunity				
Social influences	*Interdisciplinarity* *Relationship*	Lack of engagement by physicians from other disciplines (outpatient care setting)	#16 ‘Because when I sign off on the plans and distribute them, I never know if they are truly accurate. Because, various specialists, someone is always making changes, and no one commits to using this medication plan – at least not the specialists. So, we are left with the medication plan.’ (HI.01.08)	**13**
			#17 ‘Yeah. And they do their thing. And we also have patients in nursing homes – I’ve told them, for example I have one patient, he’s got dementia and so on. And now the neurologist has prescribed him [unintelligible]. And then the staff at the home come and say: ‘This isn’t working.’ And I say: ‘I’m not going to touch that.’ I really have to say, that’s not the right medication for this case. But I can’t reach him, he doesn’t talk to me, nothing. So it’s… I mean, psychiatrist communication is zero – that’s what I claim, at least.’ (HI.05.01)	
			#18 ‘Wenn das Ganze nicht funktioniert oder die Strukturen dafür nicht etabliert sind, ist es einfach schwierig.’ (HI.01.03)	
	*Interdisciplinarity* *Relationship*	Involvement of other physicians in patients treatment	#19 ‘And if the psychiatrist has already prescribed it again and it just continues, then it’s difficult. Yes. But I would have prescribed it for her again as well. What else am I supposed to do in that moment? Like that.’ (HI.01.06)	**10**
	*Interdisciplinarity* *Relationship*	Lack of collaboration/awareness regarding medication safety by hospital physicians	#20 ‘I mean, when I create a new medication plan for an elderly patient with, say, twelve medications, and he goes to the hospital, they change everything. The patients come back without knowing what he is taking, and it becomes incredibly time-consuming. And also with, yeah, in the hospital they do not review all the new medications thoroughly with the patient.’ (HI.01.08)	**9**

GP: General Practitioner. COM-B: Capability Opportunity Motivation – Behaviour. TDF: Theoretical Domains Framework. I: Interviewer. P: Participant. CPG: Clinical Practices Guideline.

#### Barrier: Psychological capability

Time constraints were a pervasive issue, with 13 GPs reporting that time scarcity negatively impacted both their cognitive capacity and ability to integrate medication reviews into daily routines. Medication reviews were not systematically conducted, and deprescribing of antidepressants occurred incidentally rather than as part of structured practice (quote #2, #3). Insufficient knowledge about clinical practice guidelines (CPGs) and lack of existing CPGs related to deprescribing antidepressants, noted by 9 GPs, further hindered informed decision-making (#1).

#### Barrier: Reflective motivation

Uncertainty about when to consider deprescribing antidepressants was expressed by 9 GPs, who struggled with identifying appropriate situations for discontinuation (#4, #5). A total of 13 GPs also identified patient-related barriers, such as fears, scepticism, and emotional distress, as significant hindrances to deprescribing efforts (TDF: Beliefs about Consequences). Furthermore, 9 GPs found deprescribing antidepressants more challenging than other medications, particularly those without mental health or other symptomatic effects (#6).

#### Barrier: Automatic motivation

Tools like potentially inappropriate medication (PIM) lists, such as PRISCUS [12], FORTA ([13], n = 12), and guidelines such as the German National Disease Management Guideline on Unipolar Depression [14] were criticised by 12 GPs for being overly complex and time-consuming (#7–#9). Overall, 7 GPs reported abandoning these tools after initial attempts due to their impracticality.

#### Barrier: Physical opportunity

Heavy workloads and lack of time were cited by 17 GPs as critical barriers to deprescribing, preventing the development of routine practices or promoting a ‘deprescribing mindset’ in daily operations (#10). The absence or inadequacy of practical tools and specific guidelines for antidepressant deprescribing further compounded these challenges (#11–#14). 9 GPs felt financially undervalued, and four expressed frustration at the lack of recognition from policymakers, which discouraged additional efforts in medication review and deprescribing (#15).

#### Barrier: Social opportunity

Interdisciplinary collaboration posed significant challenges, with 13 GPs reporting inadequate engagement from specialists, including psychiatrists and cardiologists, in medication safety and deprescribing efforts (#16–#18). 9 GPs highlighted issues with patients returning from hospitals with altered medication plans that lacked explanations, leaving GPs and patients uncertain about changes (#20). Moreover, 10 GPs expressed frustration at the lack of support from other stakeholders, such as hospital physicians, in managing pharmacotherapy (#19).

### Facilitators to antidepressant deprescribing

The following section provides a detailed discussion, referencing quotes from Table 3.

**Table 3.** Facilitators to antidepressant deprescribing reported by general practitioners participating in semi-structured interviews.

TDF domain	Theme	Codes	Sample quotes	No. of GPs reporting codes
COM-B domain: Psychological Capability				**(*n* = 20)**
Mental skills (cognitive)	*Relationship* *Guidance and decision*	Communication with patient/patient involvement	#21 ‘I speak in clear sentences. I then ask if they have understood. I repeat often. And very frequently, I work with my own self-designed recommendations.’ (HI.01.02)	**17**
Knowledge	*Relationship* *Guidance and decision*	Shared decision making (when/how to describe)	#22 ‘They usually tell me, ‘Yes, I have already thought about that,’ and then explain to me why now is a good time. Or we discuss when a good time might be. So, it’s more of a safeguard. Then we think about how to proceed. Sometimes we postpone the decision, and I say, ‘Well, it might not be the right time yet, I’ll ask you again later.’ That’s how it goes. Yes.’ (HI.01.10)	**18**
			#23 ‘That actually always works quite well. That we take the time to look at the situation: how is the patient doing, what helps them, what is the patient’s wish – and then we discuss it with the family members accordingly. The benefit. That’s when it fits quite well – for deprescribing.’ (HI.04.01)	
			#24 ‘Actually, I usually ask the patient how they’re doing – mentally, whether they’re stable. Since when they’ve been taking that specific antidepressant, or what exactly the situation was. And depending on how that turns out, I offer: okay, we could try to reduce it – gradually, step by step.’ (ps_03)	
Memory, attention and decision processes	*Guidance and decision*	Established working routine	#25 ‘Our staff ensures that every new medication prescribed, especially if requested by the patient, is specifically reported to us. We don’t allow any new medication to go through the e-prescription process unnoticed, so to speak, without checking first. We take a moment to review the medication plan, ideally. For instance, if a patient receives an antidepressant from their psychiatrist and is already on five other medications, we make sure to check if there’s any potential interaction. We always do a quick cross-check in such cases.’ (HI.01.08)	**20**
COM-B domain: Reflective Motivation				
Beliefes about capabilities	*Guidance and decision*	Belief that deprescribing antidepressants is not difficult/more difficult compare to other drugs	#26 I: ‘Okay, great. Thank you. Do you find discontinuing antidepressants more difficult compared to other medications, like a statin, for example?’ P: ‘No, discontinuing them is not more difficult for me.’ (HI.01.03)	**11**
Beliefs about consequences	*Guidance and decision*	Benefit of deprescribing/interventions	#27 ‘Nevertheless, deprescribing is fundamentally good. The saying, ‘Every medication less is a good medication,’ is something, I do, I often say in various places and truly believe in.’ (HI.01.03)	**15**
			#28 ‘So I had a patient with hyponatraemia and dizziness. We noticed the dizziness first, and then found the hyponatraemia. Then she also started vomiting and so on – the combination was a bit strange. So we took blood samples and went through the medication. And then we thought, well, she’d been on that antidepressant for quite a while and we hadn’t really reflected on it, but realised we could probably stop it. Then we found out it was likely related. So we reduced it, and the hyponatraemia also resolved. That was pretty much the first ‘aha’ moment I had in that regard.’ (HI.05.01)	
		Belief in own capability towards deprescribing	#29 ‘And, as I said, you can relatively easily discontinue psychotropic drugs and diuretics, you know, it is, as I said, relatively straightforward.’ (HI.01.02)	**15**
COM-B domain: Automatic Motivation				
Reinforcement	*Guidance and decision*	Positive experience with deprescribing	#30 ‘It has always worked so far. I do it reluctantly, for example, in winter, during the darker months when patients are more likely to be in low spirits. Spring is actually a very, very good time to start or to try it.’ (HI.03.01)	**12**
COM-B domain: Physical Opportunity				
Environmental context and resources	*Guidance and decision*	Integrated/personalised practice managment software	#31 ‘Essentially, for me, it would be / So if I just go into my system, into the / into the medication plan, there / the potential interactions already light up there, in the plan, meaning in the compilation of the medications. I immediately get a / I don’t have to do an interaction check labouriously and then read through all the lists. Instead, it directly / shows the / the two medications that might cause an issue, with a potential adverse drug reaction and a suggestion for reduction. I would find that kind of digital solution fantastic.’ (HI.01.08)	**16**
	*Guidance and decision*	Digital/online tool	#32 ‘So, it is like this: I have a digital practice, which means my medication tool in the software warns me when I prescribe things that have interactions. There are different levels of warnings from yellow, orange, to red, and I can still prescribe if I choose to. But I am alerted to these interactions. This is actually one of the main tools I have.’ (HI.01.10)	**13**
COM-B domain: Social Opportunity				
Social influences	*Guidance and decision*	Patients desire to discontinue antidepressant	#33 ‘Yes, well it’s, it happens that patients frequently bring it up actively.’ (HI.01.04)	**14**
	*Relationship*	Good patients’ compliance and relationship with patients	#34 ‘So, the individual trust in the person? Yes, of course, it is beneficial. If I know that I can rely on them to tell me the truth, whether intentionally or unintentionally, it is certainly a support for me.’ (HI.01.09)	**8**
			#35 ‘No, I need their trust. That only works if they’re on board. But by asking repeatedly whether they might be taking too much, some eventually change their mind and say: ‘Yes, we could give it a try.’ You can’t force it – you just have to stay open.’ (ps_01)	
	*Interdisciplinarity* *Relationship* *Guidance and decision*	Support by pharmacies	#36 ‘Yes, that is the interesting part, because pharmacies, at least in our case, very often conduct interaction checks. And pharmacies often know more than we do regarding the patient’s medication. Because they also gather medications prescribed by specialists, and then interaction feedback quickly comes from the pharmacy, for example. Or feedback like, ‘Hey, they have been taking this for very very long,’ when it comes to hypnotics and sedative medications. ‘You should perhaps discuss it with the general practitioner.’ And that works very well here.’ (HI.03.01)	**12**
			#37 ‘Well, there’s the large group of pharmacists. Like for example in the Netherlands – I only know that it works really well there with the in-house pharmacies. Right. So that would certainly be a good way of checking, too.’ (HI.05.02)	

GP: General Practitioner. COM-B: Capability Opportunity Motivation – Behaviour. TDF: Theoretical Domains Framework. I: Interviewer. P: Participant. CPG: Clinical Practices Guideline.

#### Facilitator: Psychological capability

GPs highlighted effective communication skills and patient involvement as essential facilitators in deprescribing. A total of 17 GPs expressed confidence in their communication abilities, emphasising the importance of building strong relationships with patients and engaging them in shared decision-making processes (#21). When deciding when and how to deprescribe an antidepressant, 18 GPs relied on a combination of experience, intuition, and discussions with patients to determine the optimal time (#22–#24). Established routines for reviewing medications further supported deprescribing efforts, with all 20 GPs noting the value of structured workflows (#25).

#### Facilitator: Reflective motivation

Optimism and a positive attitude towards medication safety and deprescribing were identified as key facilitators. Overall, 15 GPs believed in their capability to deprescribe antidepressants effectively, particularly emphasising that reducing medications often resulted in better outcomes for patients (#27–#29, TDF: Beliefs about Consequences; #26, TDF: Beliefs about Capabilities).

#### Facilitator: Automatic motivation

A total of 12 GPs cited positive experiences with antidepressant deprescribing as a motivating factor for continuing such practices (#30). Potentially motivating and facilitating factors, as mentioned by 19 participants, were an integrated, intelligent and patient-individualised clinical decision support software or digital tools.

#### Facilitator: Physical opportunity

The availability of integrated digital tools and personalised practice management systems was a major facilitator, with 16 GPs citing features such as alerts for drug interactions as particularly helpful (#31). Additional tools like Medflex (a messaging platform), Deximed (a resource for drug interaction information), and Arriba (a tool for visualising medication effects) were mentioned by 13 GPs as supportive, albeit with room for improvement (#32).

#### Facilitator: Social opportunity

Strong patient relationships were seen as critical, with 14 GPs emphasising the importance of patient trust and compliance in supporting deprescribing (#34, #35). Patients’ willingness and desire to discontinue antidepressant therapy further facilitated deprescribing efforts (#33). Pharmacist support was another significant resource, as noted by 12 GPs, who valued pharmacists’ role in ensuring medication safety and managing drug interactions (#36, #37). Collaboration with other physicians, effective communication, and accessibility were also highlighted as supportive measures by 11 GPs.

## Discussion

### Main findings

This interview study revealed that most barriers were associated with the COM-B component Physical Opportunity (TDF domain: Environmental Context and Resources). Key barriers included time constraints, lack of practical tools, and limited collaboration with specialists and hospital physicians. Facilitators are primarily related to the Physical and Social Opportunity components, with GPs highlighting the importance of digital tools, pharmacist support, and effective communication with patients. Although the use of digital tools such as Deximed or Medflex was not systematically assessed in this study, several GPs mentioned them as potentially helpful for supporting deprescribing decisions. While such tools are not yet consistently implemented across German primary care settings, integrating familiar and accepted resources may enhance the feasibility of deprescribing interventions and better align them with GPs’ everyday practice realities.

GPs perceive deprescribing not as a separate clinical task, but as part of their broader therapeutic oversight. Decisions to reduce or discontinue antidepressants are embedded within ongoing care relationships and involve the same clinical reasoning processes as other long-term therapy adjustments. This underscores the importance of viewing deprescribing as a component of continuity of care and holistic medication management, rather than as a standalone intervention.

### Strengths and limitations

To our knowledge, this is the first study to explore antidepressant deprescribing barriers and facilitators specifically among GPs in Germany. The study’s strength lies in its diverse sample, encompassing a range of professional experiences and geographic locations, as well as its use of the COM-B and TDF frameworks for systematic analysis. This approach provides a structured understanding of behavioural influences and facilitates the identification of actionable intervention targets.

However, the study has limitations. The sample was limited to GPs in Bavaria, which may restrict the generalisability of the findings to other regions or healthcare contexts. Selection bias may have occurred, as GPs with an interest in deprescribing might have been more likely to participate. Additionally, the interview focus on barriers may have influenced participants to emphasise challenges over facilitators. Efforts were made to mitigate this by conducting pilot interviews and refining the guide through team discussions.

The difficulty of identifying barriers that GPs are either unaware of, overlook or hesitate to disclose is a well-documented limitation of qualitative interviews [15]. Social desirability bias can lead to underreporting of professional shortcomings, such as knowledge gaps and clinical uncertainties around deprescribing antidepressants, and ingrained routines and conceptions (which may be influenced by pharmaceutical representatives [16,17]) may hinder GPs from consciously reflecting on antidepressant use. Although such barriers may not have been fully captured in this study, it therefore seems prudent to include education as one component of future interventions to improve antidepressant prescribing and deprescribing practices.

### Comparison with literature

The findings align with existing literature, emphasising time constraints as a significant barrier to deprescribing in primary care settings [6,18–20]. Similar to international studies, GPs in this study reported challenges in managing heavy workloads and brief consultation times, which often reinforced the continuation of antidepressant use [6,20,21]. Uncertainty about when and how to deprescribe, frequently described in the literature [6,18,21], was also observed in this study, with GPs more relying on intuition rather than on structured guidance.

Lack of financial incentives, collaboration challenges with specialists and limited interdisciplinary support were notable barriers, echoing findings from previous research [5,6, 18,20–22]. Pharmacists, however, were recognised as valuable allies, consistent with international evidence highlighting their role in deprescribing efforts [5,23]. A key difference from other studies was the relatively high confidence expressed by GPs in their communication skills and ability to involve patients in shared decision-making, which facilitated deprescribing discussions. Also in contrast to previous studies, limited availability of non-pharmacological treatment options (e.g. psychotherapy) was not identified as a relevant deprescribing barrier in this study [18]. While patient-related concerns such as fear of relapse or anticipated resistance were highlighted in studies according to the systematic review by van Leeuwen et al. from 2024 [6], our findings place these concerns within a broader context of general practitioners’ longitudinal therapeutic responsibility. In contrast to the review, which presents deprescribing primarily as a discrete clinical challenge, our study suggests that German GPs tend to embed antidepressant deprescribing within ongoing care processes, often linking it to medication safety, long-term planning, and trust-based patient relationships.

## Implications

The findings offer important implications for developing targeted interventions to support antidepressant deprescribing in German primary care. Interventions should address time constraints through streamlined workflows and practical decision-support tools. Enhancing interdisciplinary collaboration and providing financial incentives for deprescribing efforts could further support GPs in overcoming systemic barriers [23,24]. Tailored training programs and improved integration of digital tools into practice management software, which support opportunistic identification of potential deprescribing indications, may also facilitate effective deprescribing of antidepressants [24–26]. In Germany, there is currently no standardised digital interface connecting primary care, hospital systems, and other healthcare institutions. As noted by several participants, GPs often rely on discharge letters to obtain information about medication changes, which frequently vary in quality and timeliness. In some cases, patients themselves remain the primary source of information following hospital stays. Improved digital integration across sectors could significantly enhance the safety and continuity of pharmacotherapy.

Although not a primary focus, several participants described deprescribing as a natural part of their ongoing responsibility for patient care. This reflects the embeddedness of deprescribing within the longitudinal relationships and therapeutic continuity characteristic of general practice. This may offer an additional perspective for understanding how GPs approach the discontinuation of antidepressants in routine care.

## Conclusions

This study identified key barriers and facilitators influencing antidepressant deprescribing among GPs in Germany, contributing valuable insights into the behavioural factors shaping this process. Addressing time constraints, enhancing interdisciplinary collaboration, and integrating decision-support tools into clinical practice could facilitate deprescribing. Our findings inform the design of a complex intervention to promote appropriate deprescribing in the German general practice setting.

## Availability of data and supplementary material

The main data generated during this study are included in this published article. Supplementary material (i.e. coding tree, summaries, etc.) can be accessed via https://doi.org/10.6084/m9.figshare.28330160. Further data can be provided upon request to the corresponding author via mail: jochen.vukas@med.uni-muenchen.de.
